# Clinical application of kampo medicine (rikkunshito) for common and/or intractable symptoms of the gastrointestinal tract

**DOI:** 10.3389/fphar.2015.00007

**Published:** 2015-01-30

**Authors:** Kazunari Tominaga, Tetsuo Arakawa

**Affiliations:** Department of Gastroenterology, Osaka City University Graduate School of Medicine, Osaka, Japan

**Keywords:** general malaises, gastroesophageal reflux diseases, functional dyspepsia, gastrointestinal motility, acotiamide

## Abstract

Gastroenterological reflux disease and functional dyspepsia are usually treatable using Western medical practices. Nonetheless, some cases present with intractable symptoms that are not amenable to these therapies. Treatment with kampo, a traditional Japanese medicine, recently has been proposed as an alternative therapy for use in combination with the Western practices. In general, traditional Japanese medicines have been used empirically for intractable symptoms correctively designated as “general malaises.” Accumulating lines of evidence, including basic and clinical researches, have demonstrate detailed mechanisms where traditional Japanese medicines exert pharmacological action to improve symptoms. Therefore, traditional Japanese medicines have been gaining use by various medical doctors as the specific modes of pharmacological action are recognized. This review covers both the pharmacological functions and the clinical efficacies of rikkunshito for use in treating disorders of the gastrointestinal tract.

## INTRODUCTION

In Japan, an education in the practice of traditional Japanese medicines was not popular in the standard educational system until recently. This circumstance may be due to the fact that the detailed mechanism(s) of the pharmacological action of traditional Japanese medicines, including rikunshito, remains unknown. Such mechanism(s) have been difficult to define, in part because traditional Japanese medicines usually incorporate a variety of components. As a result, many traditional medicines have been used empirically that is, for the treatment of specific symptom. For instance, the traditional Japanese medicine, kampo, often is prescribed (typically as combination therapy with modern Western practices) for patients with medically unexplained physical symptoms or intractable gastrointestinal (GI) symptoms. This patient group represents a distinct population compared to that of patients with more typical and milder symptoms. Therefore kampo therapy, like many traditional Japanese medicines falls within the field of experience-based medicine, in contrast to that of evidence-based medicine. However, evidence for the efficacy of traditional Japanese medicines, particularly for rikkunshito, continues to accumulate (Tominaga and Arakawa, 2013). Hence, this review covers both basic and clinical evidences of rikkunshito, including pharmacological functions and clinical efficacies for the treatment and management of disorders of the GI tract.

## WHAT IS RIKKUNSHITO?

**FIGURE 1:**
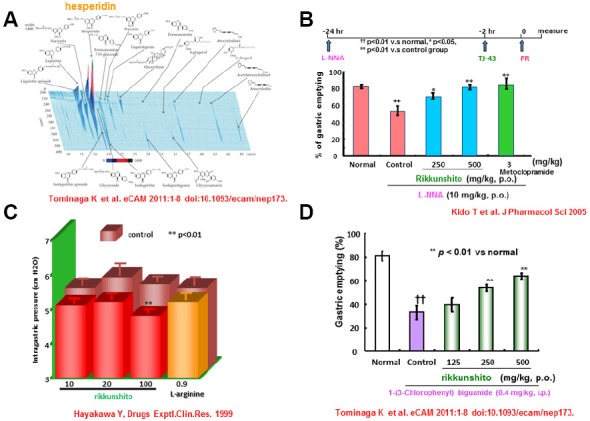
**Pharmacological actions of rikkunshito on gastric motility. (A)** Three dimensional analysis for rikkunshito by high performance liquid chromatography. **(B)** Effect of Rikkunshi-to on the decrease of the gastric emptying rate induced by L-NNA. **(C)** Effect of Rikkunshi-to on adaptive relaxation in isolated guinea pig stomachs. **(D)** Improvement in the 5-HT3 agonist [1-(3-chlorophenyl)biguanide]-induced delay in gastric emptying by rikkunshito.

Rikkunshito first appeared in the Japanese literature in the 1500s. This agent has since become one of most famous and prescribed medicines among the traditional medicines in Japan. Rikkunshito is composed of 8 constituents: *Glycyrrhizae* radix, *Zingiberis* rhizoma, *Atractylodis lanceae* rhizoma, *Zizyphi* fructus, *Aurantii nobilis* pericarpium, *Ginseng* radix, Pinelliae tuber, and *Hoelen* (Hattori et al., 2010). In recent times, analysis of the components of rikkunshito has been actively performed using high performance liquid chromatography. This work revealed that rikkunshito contains several ingredients such as liquiritin apioside, liquiritin, liquiritigenin, isoliquiritin apioside, isoliquiritin, isoliquiritigenin, glycyrrhizin, narirutin, and hesperidin (Endo et al., 2014; Figure 1). Recently, hesperidin was shown to have the highest potency (among these components) in a model of gastric emptying delay. Together, these findings support the broad use of rikkunshito for patients with dysfunction of the upper GI tract, including abdominal bloating, discomfort, nausea, and anorexia.

## BASIC PHARMACOLOGICAL FUNCTION OF RIKKUNSHITO: PROTECTIVE EFFECT ON MUCOSAL INJURIES AND PROKINETIC EFFECT ON GI MOTILITY

Gastric mucosa is sometimes injured by various factors such as ethanol, non-steroidal anti-inflammatory drugs, systemic stress, or *Helicobacter pylori* infection (Watanabe et al., 2000; Hamaguchi et al., 2001; Takeuchi, 2012). Proton pump inhibitors (PPIs), histamine type 2 blockers, or mucoprotective agents are effective for treating such acute mucosal injuries (Arakawa et al., 1998; Yanaka et al., 2007). Thus, Western medical practices are sufficient for the treatment of such conditions. In addition to these conventional drugs, traditional Japanese medicines, including rikkunshito, also can protect against acute gastric mucosal injuries. Traditional Japanese medicines, including rikkunshito, are equality effective in treating ethanol-induced acute gastric mucosal injuries, through the amelioration of mucin content decrease of the gastric mucosa (Goso et al., 1996). Such effect is also partially mediated by nitric oxide (NO) but not by prostaglandins or sulfhydryls (Arakawa et al., 1999). Therefore, rikkunshito exerts protective effect against acute mucosal injuries in the rat model. However, there is no definitive evidence for this pharmacological function of rikkunshito under the acute and chronic conditions of the human stomach.

Gastric motility, a physiological function of the GI tract, generally consists of three phases: reservoir, churning, and emptying. The total coordination of these three phases is considered essential to healthy GI physiology. Among the three phases needed for gastric motility, gastric accommodation is considered to be the most important. Gastric accommodation provides the reservoir function, which originates in the proximal stomach and requires the activity of the neuronal transmitter NO (Desai et al., 1991; Uno et al., 1997). Rikkunshito is known to promote gastric accommodation in isolated guinea pig stomach (Hayakawa et al., 1999). Rikkunshito also relaxes the smooth muscles at the fundus portion of stomach isolated from diabetic neuropathic rats with gastric dysmotility (Kito and Suzuki, 2010). The observed pharmacological functions also are induced by L-arginine, a substrate of NO synthase that is also a component of rikkunshito. Like prokinetic agents, rikkunshito counteracts the attenuation of gastric dysmotility resulting from inhibition of NO synthesis (Hayakawa et al., 1999; Kido et al., 2005). Furthermore, rikkunshito attenuates the delay of gastric emptying mediated by the serotonin (5-HT) type 3 receptor, but does not counteract the delay caused by activation of dopamine receptors (Tominaga et al., 2009). Together, these findings indicate that rikkunshito can ameliorate gastric dysmotility mediated by NO or 5-HT pathways (Kido et al., 2005; Tominaga et al., 2009). Interestingly, rikkunshito enhances endogenous ghrelin levels in plasma, and regulates esophageal and gastric motilities (Takeda et al., 2008; Yakabi et al., 2010). Ghrelin is known to have a strong orexigenic effect (Kojima et al., 1999; Nakazato et al., 2001) and enhance GI motility (Fujino et al., 2003; Inui et al., 2004; Yang et al., 2014). It also has reported that rikkunshito attenuates impairment of GI motility via dysfunction of ghrelin signaling in a rat model of gastroesophageal reflux disease (GERD; Nahata et al., 2012). More recently, Nahata et al. (2014) extended this work to a model of gastric dysmotility. Those authors reported that acute restraint stress caused plasma acylated/desacyl ghrelin imbalance and gastric dysmotility in rat, and that exogenously administered acylated ghrelin relieved the gastric dysfunction caused by this stress. Rikkunshito was shown to enhance endogenous ghrelin signaling and to provide similar relief of stress-induced gastric dysmotility (Nahata et al., 2014). In a model of reflux esophagitis (RE), rikkunshito also inhibited the activation of ERK1/2 and decreased substance P and calcitonin gene-related peptide (CGRP) levels in Th8–10 dorsal root ganglia (Kondo et al., 2014). This finding may indicate that rikkunshito inhibits afferent neuronal activity associated with visceral pain, ameliorating decreased voluntary movement in this model. Together, these research data suggest that rikkunshito may be a promising drug for the treatment of GERD and functional dyspepsia (FD) in the clinic; this medicine’s activity presumably is mediated by its pharmacological effects on esophageal and gastric motilities (Figure 1).

## CLINICAL EFFICACIES OF RIKKUNSHITO FOR UPPER GI SYMPTOMS

### TREATMENT WITH RIKKUNSHITO FOR GERD

The prevalence of obesity and metabolic syndrome in Japan continues to rise. At the same time, the rate of *H. pylori* infection in Japan is on the decrease. Under these circumstances, the prevalence of GERD is expected to increase in Japan, as has been seen in Western countries (Fujiwara and Arakawa, 2009; Fujikawa et al., 2012). The reflux of gastric contents, consisting primarily of gastric acid, is reported to be principal source of the pathogenesis of GERD (Vakil et al., 2006). Additionally, transient lower esophageal sphincter relaxation (TLESR) also is recognized to be critical to the pathogenesis of GERD (Hershcovici et al., 2011). Further GERD-associated factors include esophageal dysmotility, hypersensitivity to gastric acid or bile acid within the lower part of the esophagus, and gastric dysmotility affecting gastric accommodation and emptying. PPIs have been established as among the most effective drugs for treatment of GERD (Coté and Howden, 2008). However, other drugs regulating the esophageal motor functions also may prove useful in GERD treatment. Based on the above-described data on rikkunshito, the agent is a potential candidate treatment for patients with GERD, and so has been the subjects of clinical research. In a study that used pH multichannel intraluminal impedance monitoring, rikkunshito increased esophageal clearance and decreased esophageal acid exposure time, yielding relief of nausea symptoms in children with GERD (Kawahara et al., 2014). This traditional medicine also has been reported to relieve heartburn and acid regurgitation (Hiyama et al., 2008).

In our own clinical work, we often encounter adult patients with PPI-refractory GERD, although PPIs typically are very effective for treating this disease. Anecdotally, PPI-refractory GERD often is observed in patients lacking erosions of the esophageal mucosa (non-erosive reflux disease, NERD). In the GERD 4 study (a randomized, parallel comparative study of PPI-refractory patients with GERD), combined treatment with rikkunshito and standard-dose rabeprazole showed an efficacy similar to that of double-dose PPI treatment (Tominaga et al., 2012). In a subsequent study (G-PRIDE: A randomized, placebo-controlled, double-blind clinical trial of rikkunshito for patients with NERD refractory to PPI), rikkunshito improved psychological quality of life (QOL) compared to the placebo control (Tominaga et al., 2014). In addition, among secondary endpoints, significant efficacy of rikkunshito was observed for acid-related dyspeptic symptoms, especially among non-obese patients, women, and the elderly. Subclass analysis of the elderly PPI-refractory NERD patients in the G-PRIDE study also showed that rikkunshito significantly improved total and acid-related dysmotility scores after the 8-week treatment interval compared to placebo. In addition, 8-week combination therapy with rikkunshito significantly improved symptoms of abdominal bloating, “heaviness” of the stomach, sick feeling after meals, and heartburn after meals (Sakata et al., 2014). Beyond standard symptoms, patients with GERD also are known to exhibit extra-esophageal symptoms. Interestingly, rikkunshito has been shown to be effective for patients with globus sensation, an effect mediated by attenuation of delayed gastric emptying (Tokashiki et al., 2013).

**FIGURE 2:**
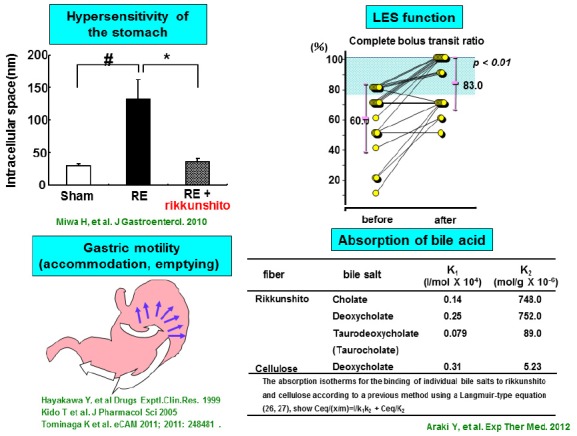
**Pharmaceutical efficacies of rikkunshito for GERD treatment**.

On the other hand, rikkunshito also has activities distinct from prokinetic functions. In a rat RE model, rikkunshito was shown to promote tight junction protein formation and to contribute to the repair of the dilation of intercellular spaces in the epithelial mucosa (Miwa et al., 2010). As with gastric acid, bile acid reflux is important for mucosal hypersensitivity in the pathogenesis of PPI-refractory GERD. Rikkunshito is reported to exhibit potent and differential absorption of bile salts (Araki et al., 2012). Notably, these findings suggest that rikkunshito may attenuate mucosal hypersensitivity to gastric acid or bile acid. These distinct pharmacological functions of rikkunshito may be a mechanism for relief of GERD symptoms in PPI-refractory NERD patients. Currently, long-term maintenance and enhanced therapies are recommended as future therapeutic regimens for PPI-refractory GERD patients (Kinoshita et al., 2012). Based upon these evidences, treatment regiments incorporating rikkunshito may be beneficial to GERD patients, particularly for PPI-refractory cases. (Figure 2).

### TREATMENT WITH RIKKUNSHITO FOR FD

Functional dyspepsia presents as symptomatic gastritis. Pathogenesis of this disease is similar to that of chronic gastritis associated with *H. pylori* infection. Therefore, FD recently has been categorized as a disease distinct from chronic gastritis, and therefore has been classified as a member of the functional gastrointestinal disorders (FGIDs). According to the 2006 Rome III criteria (Drossman, 2006), the diagnosis of FGIDs is indicated in FD patients exhibiting any of four symptoms (epigastric pain, epigastric burning, postprandial fullness, or early satiety) originating from the gastroduodenal tract without evident organic injuries. These symptoms often persist even after a variety of treatments, decreasing the QOL in patients with FD (Kinoshita et al., 2011). The pathophysiology of FD can reflect disorders of GI motility (Tominaga et al., 2008), acid secretion (el-Omar et al., 1995; Iwakiri et al., 2013), visceral hypersensitivity (Farré et al., 2013), *H. pylori* infection (Mönnikes et al., 2005), psychological factors (Tominaga et al., 2007; Ochi et al., 2008), and imbalance of the autonomic nervous system (ANS; Park et al., 2001). Of these factors, GI dysmotility has been most frequently associated with the pathogenesis of FD; however, this emphasis may reflect the relative ease of evaluating the physiological function of the GI tract in patients with FD. Gastric motility, notably its delay, has been a focus of evaluation in the pathogenesis of FD (Brun and Kuo, 2010). However, total coordination of gastric motility needs to be further elucidated for its potential role in the pathogenesis of FD. Indeed, disorder of gastric accommodation may cause epigastric discomfort, early satiety, and bloating in patients with FD (Bredenoord et al., 2003). Previously, we have reported that the impairment of gastric accommodation is associated with a delay in gastric emptying, resulting in dyspeptic symptoms (Tominaga et al., 2008). Recently, the high efficacy of acotiamide was demonstrated for treatment of dyspeptic symptoms in FD patients, an effect mediated by improvement of gastric accommodation (Matsueda et al., 2012; Kusunoki et al., 2012). Based on the results of various clinical trials, only acid suppressant drugs, prokinetic drugs, and eradication therapy for *H. pylori* have (until recently) been strongly recommended in Japan for treatment of FD. In this context, rikkunshito has been reported to ameliorate the delay in gastric emptying in patients with non-ulcer dyspepsia (NUD; Tatsuta and Iishi, 1993). The same report indicated that rikkunshito also alleviated various upper GI symptoms in patients with NUD (Tatsuta and Iishi, 1993). Moreover, rikkunshito has demonstrated efficacy for attenuation of impaired gastric accommodation and gastric motility (Kusunoki et al., 2010). Separate from stress’ effects on gastric motility in the pathogenesis of FD, physical and psychological stresses often cause gastric hypersensitivity to stimulation by mechanical balloon distension. Stress-induced gastric hypersensitivity and/or changes in gastric wall tone (as assessed by gastric barostat method) were relieved by rikkunshito (Shiratori et al., 2011). In a separate human study comparing rikkunshito and domperidone, rikkunshito improved symptoms of patients with FD and increased plasma ghrelin levels (Arai et al., 2012). Rikkunshito was shown to ameliorate delayed gastric emptying in severely handicapped patients (Kawahara et al., 2009) and to improve gastric myoelectric activity in post-operative dyspeptic children after GI surgery (Yagi et al., 2004). These findings suggest that rikkunshito affects some inflammatory and/or neuroendocrinal mediators as well as gastric sensorimotor function, and improves dyspeptic symptoms of FD. Apart from FD, GI symptoms sometimes occur after endoscopic treatment. Combination therapy with rikkunshito and PPI after endoscopic submucosal dissection alleviated abdominal pain symptoms (Uehara et al., 2013). Recently, a randomized clinical trial using rikkunshito was performed for FD. Eight-week treatment with rikkunshito was tended to be effective for FD, as determined by global patient assessment (Suzuki et al., 2014). Thus, rikkunshito is a potential candidate for the clinical treatment of FD.

In conclusion, rikkunshtio is considered “complementary” (non-mainstream) medicine for treatment of GI diseases such as GERD and FD. However, rikkunshito frequently is prescribed for patients with diverse GI tract disorders. Combination treatments using modern Western and traditional Japanese medicines should be considered, especially in patients presenting with intractable symptioms, including general malaises.

### Conflict of Interest Statement

The authors declare that the research was conducted in the absence of any commercial or financial relationships that could be construed as a potential conflict of interest.
